# Editorial

**Published:** 1876-02

**Authors:** 


### Editorial.

Our Library.—In this number of the Journal and Examiner we give a partly full account of the opening of the Library of the Medical Press Association, an event which must interest the whole profession of the Northwest.

The Medical Libraries of Chicago have never been extensive, and in the great fire of 1871, a number of good private collections of books were lost, and since then have not been fully replaced.

Prior to 1871, Cook County Medical Society had collected a small library, amounting, we believe, to about two hundred volumes. This was probably the first attempt made in Chicago to form a public Medical Library, and almost as soon as begun the collection was burned up. The officers of the Academy of Science have generously given the Medical Press Association, room for their Library in their fire-proof building, at 263 Wabash Avenue.

It is hardly necessary to urge the need of a public Medical Library. Without it, extensive research is impossible, and progress in the science of medicine, if not entirely prevented, is certainly very much retarded.

The extensive range of books required for thorough research, is very seldom afforded by private libraries. We know of no collection of books in Chicago, at all adequate to the demands of the professional scholar. Private libraries, in this new country, are the creation of a few years growth only, and intended to supply references for the practitioner, whose time is largely occupied with the active duties of his profession.

The books, therefore, are, for the most part, recent text books, and a very limited number of medical periodicals, which serve to complete the literary resources of most physicians. However extensive the collection, this will be its general character. In its place, this class of works is indispensable, but we have arrived at a stage of professional advancement, when this kind of a library is not sufficient. We need one containing, as nearly as possible, all the ancient, mediæval, modern and current literature of the profession.

It is hardly practicable to have extensive and varied collections, except in public libraries. In our private collections we have one of the elements required to make up this variety. All those of us who have been long occupied in the profession, possess copies of a few old books that are becoming rare. They are scattered about; one or two being in possession of each of a large number of the practitioners of the city and country, rotting on shelves, because never consulted by their possessors. Having been long superseded by modern works, they are practically valueless in private libraries as books of reference. As curiosities, we may treasure up such works as those of Hippocrates, Galen, Celsus, etc., among ancient authors; or even the modern writers, as Cullen's First Lines, Good's Study of Medicine, Dewees' Midwifery, Eberle's Practice, Fordyce on Fevers, Bell & Stokes' Practice, etc., all popular text books thirty years ago, but we would not at present think of relying upon their teaching in practice.

In our private libraries we must have the text books of recent date.

If medical men would send the kind of old books above mentioned to the public library, they would be useful to the whole profession, because accessible to all, and instead of being able to enjoy the benefit of only one or two old books, we would have the use of a large number.

It is not too much, then, to request the physicians of the city and the whole Northwest, to send such books as lie idly upon their shelves, to the library of the Medical Press Association for safe keeping. Our librarian will write in them the names of the donors, the date, and other items calculated to identify the gift, and give due credit for the bestowal.

The resources of the organization make the accumulation of contemporaneous literature an easy matter. The list of exchanges received for the JOURNAL AND EXAMINER, now numbering nearly one hundred periodicals, and increasing every month, while it affords the profession extensive facilities for keeping abreast of the times, will furnish the shelves of the library with over one hundred volumes annually. Add to this the reception of reviews of all American and many foreign medical books and pamphlets, and it will be seen that our library must grow. We have already been placed under obligations to several physicians in the city for donations of books and volumes of popular periodicals for several years back, and have the promise of a still greater number.

Will the whole profession of the city and neighborhood join us in this enterprise? If they will, the formation of a large and useful library will be the work of but a very few years.

---

THE *Bouffe* IN MEDICINE.—The *New York Nation* of Dec. 23, 1875, contains an article which illustrates, in a striking manner, the introduction into politics of the central idea of the comedies of the *opéra bouffe.* The complete inversion of the moral world and the social institutions handed down to us from the past, as exhibited in the *Grande Duchesse* and the *Belle Helène*, are mirrored in the pool of New York politics, whose slimy depths have been exposed to the eye of the world.

But the portraitures of the *bouffe* characters can be detected as well in certain circles in Chicago.

For example, during three successive days of last month, there hung, like a pall over this city, a horrible and disgusting stench, that emanated from one of the rendering establishments on the outskirts of the town. The impalpable and nauseating effluvium entered the nostrils of four hundred thousand people. They ate it with their chops at breakfast, drank it in the evening cup of tea, inhaled it alike in the parlor and bed-room. More than this. It filled hundreds of sick chambers with a revolting suggestion of the charnel-house, and poisoned the air respired by thousands of delicate infants struggling for existence against the other disadvantages of city life. Whose business was it to abate this nuisance that revisits the city ever and again with a recurring periodicity? Not the business of the Board of Health, certainly. It is true that the salaries of these officials are paid out of the pockets of the sufferers. It is true that they report, periodically, the abatement of a certain number of nuisances, while this great head and front of all nuisances obtrudes itself persistently upon the public. Is it, also, true that the proprietors of these rendering establishments occasionally see the officials of the Board of Health, and that the latter are also on such occasions "seen"?

The reason for all this lies in the fact that the Board of Health of Chicago rests upon a political basis, from its august head to its petty policeman. No young medical aspirant, with ambition and ability, need ever hope to obtain official connection with it, by acquiring a knowledge of sanitary science. The medical profession of Chicago is no more responsible for its organization, than for the conduct of a pork-packing establishment. Intimate acquaintance with the "short-haired" element—influence with "the Bosses" and "the Boys"—this is the sole avenue of ingress. This is the *bouffe* stage, gentlemen, where, before the foot-lights, *Prince Paul* and *General Boum* act their droll parts for their own pecuniary profit.

Another illustration of this principle, is to be found in the recent exposure of the officials connected with the Cook County Insane Asylum—an exposure made by a committee of medical men appointed by the Chicago Society of Physicians and Surgeons. In this institution nearly three hundred insane patients are collected. Surely, one would think, the man who is charged with the professional care of its inmates, is an expert in all the forms of mental and nervous disease—a man who has at least acquired some reputation as a skillful and judicious practitioner. By no means. There was reason for thinking the physician of this asylum had never even received a degree in medicine. The assistant physician was found to have acquired all his lore in one course of lectures attended in this city. The warden, an official superior to both of those just named, was found to be a local politician, the retention of whose position depended upon his party affilations. This, gentlemen, is the result of the *bouffe* principle—the result of an inversion of the rules by which men are guided who do not perform on the comic stage.

One of the leading milk-dealers of our city has long endeavored to secure a rigid system of milk inspection in the interest of those who desire to supply a perfectly pure article for city consumption. There is probably not a respectable physician in Chicago, unconnected with any of its various boards, who does not consider such a measure absolutely essential to the comfort and welfare of the community. Yet all efforts in this direction have proved utterly futile. It is because, gentlemen, they do not do these things in the *bouffe* opera.

All this will be remedied in time. The men who have secured their positions by political influence will be in turn supplanted by men who possess a greater influence. But the medical profession of Chicago, exerting its authority through the committees of its medical societies, has already made itself felt before grand juries and judges and the public. It has tasted of power, and, in accordance with a natural law, will, in the end, exert that power to its fullest extent.

Meantime the Offenbachian doctors will exhibit their travesty of the medical profession to the general public, surrounded by their motley short-haired acquintances. On with the play ; the curtain will fall the sooner !

We notice in the British *Students' Journal and Hospital Gazette*, of Nov. 6, a sharp censure of the medical schools of England. The inefficiency of instruction in the schools is deplored, and the prevalent habit of teaching almost exclusively by lectures is strongly condemned. "From 40 to 80 per cent. of their students are 'plucked' at the examinations of the Royal college of surgeons and other examining bodies." The students are defended as not being either lazy or deficient in brain power; the whole trouble is with the teachers; the zeal of the students is thrown away for "want of a proper guide to direct their studies"; "the cut-and-dried lecture that has done duty for successive generations of students should be dispensed with"; "the demands of science and of the examination room are, demonstrations."

One reason given for so great a per centage of failures is, that, while examinations are "more practical than formerly, the mode of education remains pretty much the same."

We are surprised at this. We had supposed the instruction given to students in England was much more thorough and practical than in our own country, and especially that it was the examiners, not the teachers, who were fossilized.

Perhaps, nevertheless, the critic is right. We most heartily agree with him, however, that the formal lecture through important, is not most important. More instruction should be given by recitations; there should be more of demonstration and more actual observation. The lecture is a great relief to the monotony of continuous reading, and adds greatly to the ability of any one to acquire knowledge. He who hears told the thing he has read, in a different fashion perhaps, retains it doubly well; the lecture is, as a means of education in medicine, a great addition to that of reading. The actual observation of a thing as a phenomenon; the working out of an experiment or a demonstration, and, more than this, the obligation to tell about it, are a great step in advance of both reading and hearing. That knowledge which is gained through the eye and by actual experience, is easiest acquired and is impressed upon us most indelibly.

The medical colleges in America will have inaugurated a grand reform, when, even if they have fewer or no more lecturers, they have many more demonstrators and tutors and drill-masters. The number of the latter should be at least equal to that of the former—it is a question whether they should not be more numerous.

---

We recommend to an early and earnest consideration of the faculties of our medical colleges, the pointed remarks Dr. Gross makes in his "History of Amer. Med. Literature" on the subject of *Medical Theses*. "Of this variety of medical literature," he says, "our colleges have huge piles, for every spring large additions are made to their archives, usually badly written, not unfrequently ungrammatical, generally devoid of scientific information, and of no use to anybody, for it is not too much to say that not one in fifty affords the slightest evidence of competency, proficiency or ability in the candidate for graduation. Often, indeed, they are not composed by him; and occasionally they are plagiarized, or copied, it may be *verbatim*, from such books as are within his reach, if not actually from the works of his preceptors. Happily for the credit of the schools, few of these productions find their way into print. * * It would be well if, on the birthday of American Independence, a bonfire could be made of this trash, as it exists, without exception, in all our medical schools; and it is devoutly to be wished that the regulation which prescribes the presentation of the inaugural dissertation were abolished." Will the colleges free themselves of this nuisance?

